# Profile of macular ganglion cell-inner plexiform layer thickness in healthy 6.5 year- old Swedish children

**DOI:** 10.1186/s12886-020-01601-y

**Published:** 2020-08-12

**Authors:** Urszula Arnljots, Maria Nilsson, Ida Hed Myrberg, Ulrika Åden, Kerstin Hellgren

**Affiliations:** 1 Department of Clinical Neuroscience, Karolinska Institutet, Stockholm, Sweden; 2 Department of Clinical Neuroscience, Unit of Optometry, Karolinska Institutet, Stockholm, Sweden; 3 Department of Women’s and Children’s Health, Karolinska Institutet, Stockholm, Sweden

**Keywords:** Optical coherence tomography (OCT), Children, Ganglion cell-inner Plexiform layer (GC-IPL), Normal values, Inter and intraocular symmetry

## Abstract

**Background:**

The purpose was to study the macular ganglion cell- inner plexiform layer (GC-IPL) thickness in healthy 6.5 year- old Swedish children using Optical Coherence Tomography (OCT) and to study topography symmetry within eyes and between eye pairs.

**Methods:**

A total of 181 eyes of 92 healthy children (39 girls, 53 boys) aged 6.5 and serving as a term-born control group in the Extremely Preterm Infants in Sweden Study (EXPRESS), were examined with Cirrus HD-OCT. Main outcome measures were average and minimum values of GC-IPL thickness of the device’s predefined macular sectors. Single sectors, combined sectors defined as superior and inferior hemispheres and temporal and nasal sectors were evaluated. Intra-individual GC-IPL thickness between eye pairs was analyzed. Visual acuity, refraction and general cognition were assessed and correlated to GC-IPL outcome.

**Results:**

Eighty-five children completed the OCT examination and 155 out of 181 scans (86%) were analyzed. The mean average GC-IPL thickness was 85.9 μm (± 5.3; 5th and 95th percentiles were 76.0 and 94.6 μm). The mean minimum GC-IPL thickness was 83.6 μm (± 4.9; 5th and 95th percentiles were 75.4 and 92.3 μm). The difference in thickness between nasal and temporal sectors and between superior and inferior hemisphere sectors were less than 2 μm. The difference between average GC-IPL thickness and minimum GC-IPL thickness was 2.3 μm (± 1.9; 5th and 95th percentiles were 0.0 and 6.0 μm). The difference between the thickest and thinnest sector within eye was 6.4 μm (± 2.2; 5th and 95th percentiles were 3.0 and 10.0 μm). There was a moderate correlation in the difference between the nasal combined and the temporal combined sectors within eye pairs (*p* < 0.0001, Spearman’s ρ 0.58). The average GC-IPL thickness was weakly positively correlated with SE (spherical equivalent; combined sphere and ½ cylinder) (*p* = 0.031, Spearman’s ρ 0.23).

**Conclusions:**

This study provides normative GC-IPL thickness values for healthy 6.5 year- old Swedish children. The GC-IPL thickness variations within eyes and within eye pairs are generally small. It could therefore be assumed that larger variations are sensitive markers of focal GC-IPL thinning due to damage to the primary visual pathways in children.

## Background

Optical coherence tomography (OCT) is a non-invasive and objective imaging technique that provides cross-sectional images of the retinal layers and the optic nerve [1]. Since OCT came into clinical use it has undergone several improvements. The newer, spectral domain technology (SD-OCT) offers three-dimensional, high-speed retinal imaging and thereby enables high resolution imaging less sensitive to eye movements than time domain technology-based OCT [2, 3]. Moreover, advances in segmentation algorithms have permitted measurement of individual retinal layers in the macular region [4]. The macular ganglion cell + inner plexiform layer (GC-IPL) has proven to be of interest in detection and follow-up of for instance in glaucoma [5, 6] and other optic nerve diseases: optic pathway glioma [7, 8], compressive optic neuropathies [9] and hereditary neuropathies [10, 11]. Although the reliability and repeatability have been proven to be satisfying when examining children with Cirrus SD-OCT [12] the GC-IPL parameter has not been extensively investigated for younger age groups and there is no normative database. Instead the peripapillary retinal nerve fiber layer (pRNFL) parameter has been more widely explored.

In children pRNFL thickness can be useful for detection and monitoring of glaucoma, optic nerve hypoplasia, optic neuritis and in the differential diagnosis of optic nerve swelling: elevation secondary to increased intracranial pressure (i.e., papilledema) or due to other congenital/structural anomalies (i.e., pseudopapilledema) [13–15]. Possible advantages of replacing or adding GC-IPL to pRNFL measurements are that loss of axons at the optic nerve head could be masked by swelling at an early stage. Fewer vessels in the macular region compared to the optic nerve head leads to better repeatability between measures and a reduction of artifacts [16]. Furthermore, damage to the post-geniculate visual pathway has been associated with focal GC-IPL thinning caused by retrograde trans-synaptic degeneration (RTSD) [17, 18]. These studies have also shown that GC-IPL topography predicts visual field defects more directly compared to pRNFL. Only a few previous studies report reference values for GC-IPL thickness in children [19–22]. These studies also varied in methodology and epidemiology.

## Methods

The purpose of the present study was to describe the intra- and inter ocular GC-IPL layer topography to determine normative data on thickness variations within and between eyes. This information may improve the preconditions for early identification of focal GC-IPL thinning caused by pathology affecting the primary visual pathway.

### Study population

The group of children aged 6.5 years (*n* = 94), was drawn from the Swedish Medical Birth Register and was serving as a term-born control group in a prospective follow-up study of Extremely Preterm Infants in Sweden Study (EXPRESS) as previously described [23, 24]. Included in the study were subjects born at term (gestational age; GA ≥ 37 weeks), at normal birthweights (≥2500 g), born in Stockholm County and with no systemic diseases.

### Methods/procedures

#### Optical coherence tomography

The OCT scans were performed by two examiners through undilated pupils using the Cirrus HD-OCT device (Cirrus; Carl Zeiss Meditec, Dublin, CA). Macular Cube 512 × 128 scan protocol and automated GC-IPL analysis segmentation algorithm, incorporated into the Cirrus 6.0 software were selected.

**Fig. 1:**
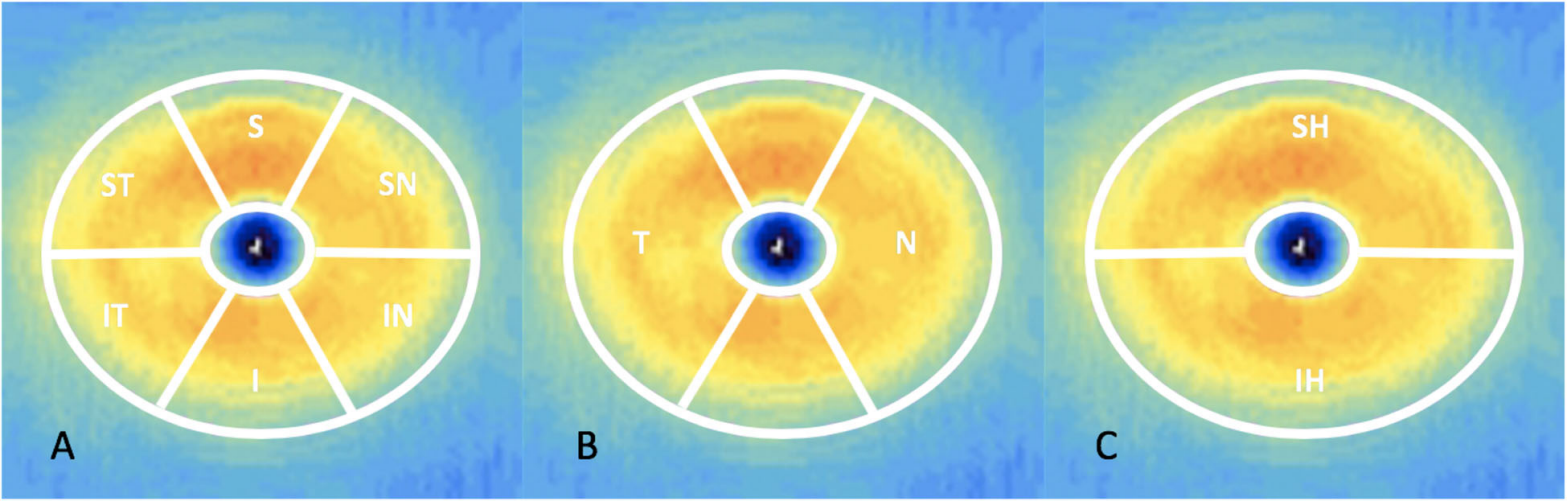
OCT image of the macula with the elliptic annulus (dimensions around the fovea)

The main outcome measures for GC-IPL thickness per eye were Average and Minimum GC-IPL (lowest GC-IPL thickness over a single meridian crossing the annulus), individual sectors (superotemporal [ST], superior [S], superonasal [SN], inferonasal [IN], inferior [I], inferotemporal [IT]]), as well as Thickest and Thinnest individual sectors. In addition, we analyzed combined sector thicknesses defined as superior (SH; ST + S + SN) and inferior hemispheres (IH; IN+I + IT) and temporal (T; ST + IT) as well as nasal (N; SN + IN) sectors (Fig. 1) [25].

The intraocular GC-IPL topography was further analyzed by subtracting Average with Minimum GC-IPL; Thickest with Thinnest individual sectors; N with T sectors and finally SH with IH. The scans were reviewed by two independent examiners (MN and UA) and those with signal strength < 6 were excluded. Further, a thorough evaluation of the remaining scans revealed a recognizable pattern of artifacts yielding segmentation errors in some of them, due to unstable fixation [26]. Those artifacts included a so-called double fovea resulting in false thinning of the GC-IPL inferior sectors [27] or false thickening of the superior sectors. Double fovea occurred due to a transient microsaccade looking upward (rotating the fovea downward into the path of the raster scan) followed by re-fixation onto the central marker. The scans with artifacts as described as above, defined as Uncomplete Scans, were not included in the final analysis. The Uncomplete Scans were compared with the scans without artifacts, defined as Complete Scans, in order to study if artifacts tend to give any general pattern/thickness deviations on group level.

*A. The six macular sectors: S = superior, SN = superonasal, IN = inferonasal, I = inferior, IT = inferotemporal, ST = superotemporal. B. The temporal (T) and nasal (N) sectors. C. The superior (SH) and inferior hemisphere (IH).*

#### Ophthalmologic assessments

Refraction was assessed with autorefractor of eyes under cycloplegia (mixture of phenylephrine 1.5% and cyclopentolate 0.85%). Visual acuity (VA) was evaluated monocularly, with habitual correction using Lea Hyvärinen symbols charts assessed at a distance of 3 m [28]. The data was presented in Snellen. Stereoacuity was measured by using TNO test.

#### Cognitive assessments

The Swedish version of Wechsler Intelligence Scale for Children–Fourth Edition (WISC-IV) was used in order to assess the cognitive ability [29]. The full-scale intelligence quotient was taken as a measure of general cognition.

Written informed consent was obtained from each subject’s parent or legal guardian. The study was approved by the Regional Ethical Review Board in Stockholm and conducted in accordance with the tenets of the Declaration of Helsinki.

### Statistical analysis

Both eyes from each patient were used for analysis. Descriptive statistics are presented as means, standard deviations and 95% confidence intervals for continuous variables, and frequencies and percentages, for categorical variables. Mean GC-IPL and 95% confidence intervals for each sector were estimated using Linear Mixed Models, with a random intercept per child, taking into account dependencies within eye pairs and between included and excluded eyes of the same child. Gender differences in mean GC-IPL were tested using Linear Mixed Models, with a random intercept per child. The GC-IPL distribution synchronicity within eye pairs was conducted with a correlation analysis between the right and left eye regarding the differences between N and T sectors as well as between SH and IH. Correlations between macula parameters and continuous variables (refraction, VA, birth weight, IQ) were calculated using Spearman’s Rank Correlation Coefficient, with cluster robust standard errors, taking into account dependencies within eye pairs. Pearson correlation was used to estimate correlation in the difference between SH and IH, and N and T, respectively, between left and right eyes within the same child. All statistical analyses were performed using R version 3.6.0.

## Results

Nighty-four children were invited to participate in the study. Two children were excluded due to missing data. In one case, a boy, imaging could not be performed due to technical problems, and in another case due to lack of cooperation. Hence, 181 eyes of 92 children (39 girls, 53 boys) completed the OCT examination. In 155 of these 181 (86%) OCT images, provided by 85 children there were Complete Scans (without artifacts) and in 26 (14%) out of 181 OCT images there were Uncomplete Scans (with artifacts). Clinical data of the 85 participants are presented in Table 1. All but one child had TNO < 120 s of arch.

**Table 1:** Clinical data of 85 study participants

	Mean (SD)	Range
Gestational age (weeks)	40.1 (1.2)	37–42
Birth weight (g)	3649.6 (430.7)	2850–4720
IQ score	105.2 (10.7)	85–130
LogMAR VA RE	0.015 (0.09)	−0.1 – 0.4
LogMAR VA LE	0.004 (0.075)	−0.2 – 0.2
Snellen VA RE	20/20 (20/100)	20/50–20/16
Snellen VA LE	20/20 (20/100)	20/32–20/12.5
SE RE (D)	1.4 (1.0)	−1 – + 6.12
SE LE (D)	1.4 (0.8)	−0.125 – + 6.25

*LE* Left eye, *RE* Right eye, *SE* Spherical equivalent, *SD* Standard deviation, *VA* Visual acuity (with habitual correction).

### The GC-IPL measurements and intraocular GC-IPL topography

**Fig. 2:**
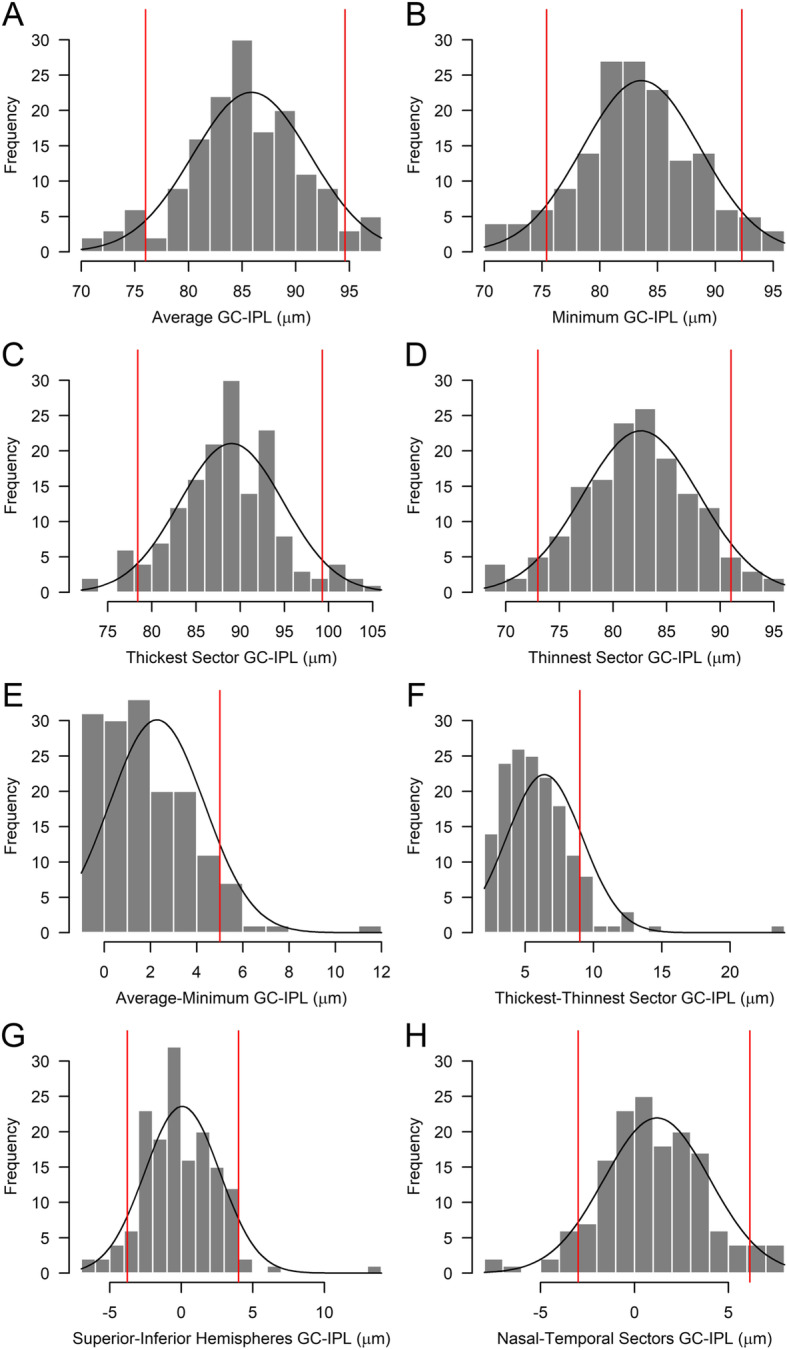
Distribution of GC-IPL thickness (histograms) compared with normal distribution (solid curve) in 155 Complete Scans. **a**. Average overall GC-IPL thickness, **b**. Minimum GC-IPL thickness, **c**. Thickest sector GC-IPL (superior, superiotemporal, inferiotemporal, inferior, inferionasal or superionasal per eye), **d**. Thinnest sector GC-IPL (superior, superiotemporal, inferiotemporal, inferior, inferionasal or superionasal sector per eye). Difference in GC-IPL thickness between: **e**. Average and minimum, **f**. Thickest and thinnest sector, **g**. Superior and inferior hemisphere, **h**. Nasal and temporal sectorial GC-IPL. Red lines indicating 5 and 95% percentiles (A, B, C, D, G, H) and 90% percentile (E, F). GC-IPL = Ganglion cell inner + plexiform layer

The GC-IPL measurements, as well as the topography as predefined intraocular sector differences of 155 eyes are presented in Table 2. The mean average GC-IPL was 85.9 (± 5.4 μm), the 5th and the 95th percentiles were 76.0 and 94.6 μm. The mean minimum GC-IPL was 83.6 (± 4.9 μm), the 5th and the 95th percentiles were 75.4 and 92.3 μm. The mean absolute difference between the Thickest and Thinnest sector was 6.4 ± 2.2, between the two hemispheres SH and IH (both directions) was 0.1 ± 2.0, between combined sectors N and T (both directions) was 1.2 ± 2.5 μm. For details of the distribution of GC-IPL measurements and intraocular differences, see Fig. 2.

**Table 2:** Macular GC-IPL thicknesses in micrometers in all eyes with Complete Scans of OCT (*n* = 155 of 85 children)

	Mean (SD; range) in μm	95% CI for the mean in μm	Percentile in μm			
			5%	10%	90%	95%
AREA AND SECTOR THICKNESSES						
Average GC-IPL Thickness (μm)	85.9 (5.3; 70–98)	(84.7–87.0)	76.0	79.0	93.0	94.6
Minimum GC-IPL Thickness (μm)	83.6 (4.9; 70–95)	(82.6–84.7)	75.4	77.0	90.0	92.3
Singel sectors						
ST	84.5 (5.6; 70–101)	(83.3–85.7)	75.0	77.0	91.0	94.0
S	86.1 (6.2; 71–103)	(84.8–87.5)	74.0	78.4	94.0	97.0
SN	87.1 (5.8; 72–103)	(85.9–88.4)	76.0	79.4	94.0	96.0
IN	86.1 (5.5; 68–99)	(84.9–87.3)	76.0	80.0	93.0	95.0
I	85.1 (5.5; 68–101)	(83.9–86.3)	76.0	78.0	92.0	94.0
IT	86.3 (5.4; 71–105)	(85.2–87.5)	77.0	79.4	93.0	96.0
Combined sectors						
SH	85.9 (5.7; 71–100.3)	(84.7–87.1)	75.1	78.6	92.7	95.9
IH	85.8 (5.2; 69.7–98)	(84.7–86.9)	76.5	79.6	92.2	95.0
N	86.6 (5.5; 71–100)	(85.4–87.8)	75.8	80.2	93.3	95.6
T	85.4 (5.4; 70.5–103)	(84.2–86.6)	76.3	78.7	92.3	94.1
Selected Single Sectors						
Thickest Sector	89.1 (5.8; 72–105)	(87.8–90.3)	78.4	82.0	96.0	99.3
Thinnest Sector	82.6 (5.2; 68–95)	(81.5–83.7)	73.0	76.0	90.0	91.0
INTRAOCULAR SYMMETRIES						
Difference between Average and Minimum	2.3 (1.9; −1 - 12)	(1.85–2.7)	0.0	0.0	5.0	6.0
Difference between Thickest and Thinnest Sector	6.4 (2.2; 2–24)	(5.90–7.0)	3.0	4.0	9.0	10.0
Difference between SH and IH	0.1 (2.0; −6.33 - 13.67)	(− 0.40–0.6)	−3.77	−2.67	3.20	4.0
Difference between N and T Sectors	1.2 (2.5; −8 - 8)	(0.66–1.7)	−3.0	−2.00	4.80	6.15

*SD* Standard deviation, *GC-IPL* Ganglion cell inner + plexiform layer, *ST* Superotemporal, *S* Superior, *SN* Superonasal, *IN* Inferonasal, *I* Inferior, *IT* Inferotemporal, *SH* Superior hemisphere, *IH* Inferior hemisphere, *T* Temporal sectors, *N* Nasal sectors.

**Fig. 3:**
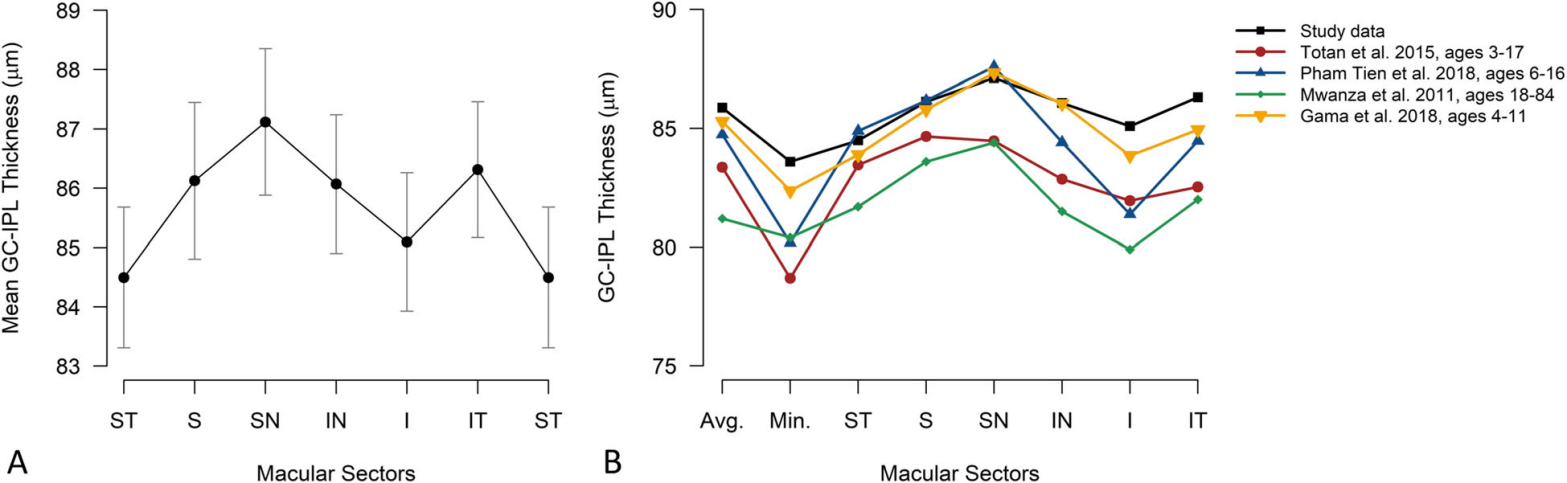
Graphic profile of mean GC-IPL thickness in six macular sectors in study population (**a**), comparison of studies reporting OCT macular GC-IPL thickness in normal pediatric population (**b**). Avg = Average, Min = Minimum, ST = superotemporal, S = superior, SN = superonasal, IN = inferonasal, I = inferior, IT = inferotemporal, GC-IPL = Ganglion cell inner + plexiform layer

The profile of GC-IPL thickness in the six macular sectors is presented in Fig. 3a and in comparison to other studies in pediatric and adult populations in Fig. 3b.

### The GC-IPL distribution symmetry within eye pairs

**Fig. 4:**
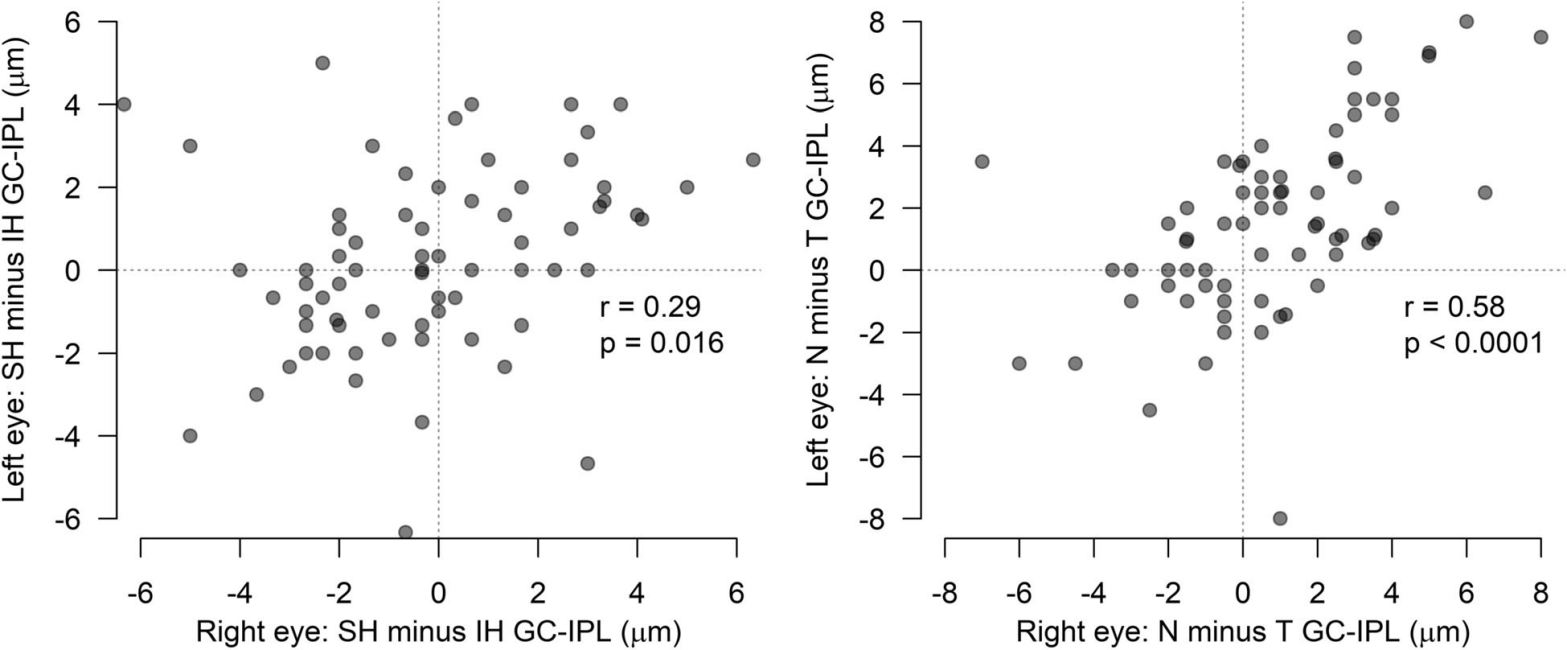
The GC-IPL distribution synchronicity within eye pairs. T = temporal sectors, N = nasal sectors, SH = superior hemisphere, IH = inferior hemisphere, GC-IPL = Ganglion cell inner + plexiform layer

Of the 85 children with Complete Scans, 70 provided scans of both eyes, enabling analyses of symmetry within eye pairs. The inter-ocular difference in average GC-IPL thickness was 1.5 (1.3, 0–6 μm). It was more common to have a thicker SH than IH, and N than T. There was a weak but statistically significant correlation in the difference between SH and IH within eye pairs (*p* = 0.016, Spearman’s ρ 0.29). There was a moderate correlation in the difference between N and T within eye pairs (*p* < 0.0001, Spearman’s ρ 0.58), for details see Fig. 4.

### Correlations between GC-IPL and clinical outcome

There was no statistically significant difference in average and minimum GC-IPL thickness between males and females (*p* = 0.30, *p* = 0.24, respectively). The average GC-IPL thickness was weakly positively correlated with SE (*p* = 0.031, Spearman’s ρ 0.23). There was no statistically significant correlation between average GC-IPL and birth weight, IQ or VA at 6.5 years (*p* > 0.05).

### Uncomplete OCT scans compared to complete scans

**Fig. 5:**
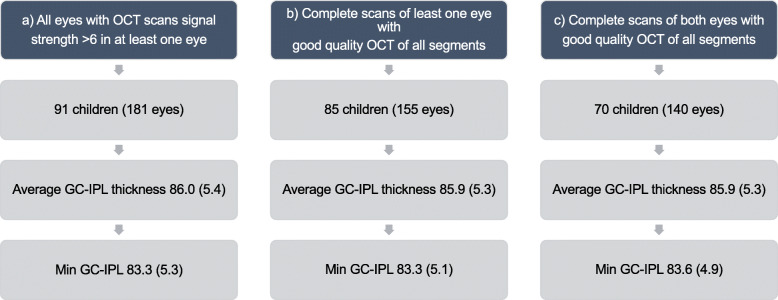
Average and minimum GC-IPL thickness in study participants stratified in subgroups depending on quality of OCT pictures. Min = Minimum, GC-IPL = Ganglion cell inner + plexiform layer. GC-IPL = Ganglion cell inner + plexiform layer, Min = Minimum

On average, the 26 images with Uncomplete Scans had thicker superior sectors than the 155 Complete Scans. The largest difference was seen in S (5.3 μm, 95% CI 3.1–7.5, *p* < 0.0001) and resulted in slightly higher average GC-IPL in the Uncomplete Scans than in the Complete Scans (mean difference 2.1 μm; 95% CI 1.1–3.0; *p* < 0.0001). None of the sectors were statistically significantly thinner among Uncomplete Scans in comparison to Complete Scans. Inclusion of Uncomplete Scans in the total analysis did not change the mean values of any OCT variable significantly. Figure 5 displays the average and minimum values of the a) 181 eyes from 91 children, with both Complete and Uncomplete Scans of at least one eye; b) 155 eyes from 85 children, with Complete Scans of at least one eye; c) 140 eyes from 70 children, with Complete Scans of both eyes.

## Discussion

The average GC-IPL thickness range, distribution and mean as well as the GC-IPL distribution between sectors seen in our study group (at 6.5 yrs) was very similar to what other have seen in groups of children aged 3–17 [19, 20] and adults [4]. In addition, we reported the “within eye” as well as “within eye pairs” GC-IPL distribution and observed that the differences are very small within individuals i.e., normal anatomy is associated with small differences in GC-IPL thickness between sectors and between eyes in the same individual. At the same time the differences in mean thickness between individuals are quite large. Therefore, it seems as important to study thickness variations between sectors and eyes on individual level as to compare with a normative database in order to increase the possibility to detect focal ganglion cell loss at an early stage of pathology.

In this study, the superonasal sector was thickest and the superotemporal sector was thinnest, but the differences were small (Fig. 3a). The same trend was observed in other studies (Fig. 3b) with similar results on a group level [19] whereas greater differences were found by *Gama* et al [20] and *Tien* et al [22]. Clinically, the attention should be paid to thickness normal profile of macular sectors, because GC-IPL thinning may be useful in detecting neurodegenerative diseases and progression [30] as well as glaucoma [5]. In addition, superior hemisphere was slightly thicker than that of the inferior, and the nasal sectors had a significantly thicker GC-IPL compered to temporal ones. This GC-IPL topography profile corresponds to *Curcio and Allen’s* histologic study, that shows greater ganglion cell density in the nasal and superior retinal regions [31]. Interestingly, the differences in the thickness between combined sectors are very small in healthy children, thus bigger differences may implicate underlying pathology.

In this study 26 OCT scans with good signal strength but with visual artifacts owing to fixation problems were excluded. Images with gaze-artifacts/segmentation error may mimic pathology by significant increased Average and Superior Sectors GC-IPL thickness. These scans should be checked and discarded, with the rescanning performed during the same visit. Interestingly, there was no significant decrease in minimum and inferior sectors GC-IPL thickness. In clinical follow-up of a small child, a thinning of superior GC-IPL over time may thus signify an improved fixation and not necessarily pathology. In a child with unstable fixation, it’s therefore important to take into account the risk of falsely thick Superior GC-IPL sectors in the baseline scans of the first examinations.

We couldn’t find an association between gender and GC-IPL thickness (*p* = 0.30) in line with *Totan* et al [19]. A mean increase in Average GC-IPL thickness with increasing hyperopia was found in this study. This result is comparable by other studies [21, 22, 32]. *Van Koolwijk* et al have previously described that cognitive functioning is associated with pRNFL thickness in healthy young individuals aged 18–39 years [33]. However, RNFL only explained a small portion of the variance in cognitive functioning. In our study group, including 6.5 year-old controls, no association between IQ and GC-IPL was found and GC-IPL does not seem to be any precise predictor of cognitive ability as estimated by WISC-IV in young healthy children.

Limitation of this study that the Cirrus Ganglion Cell Analysis does not account for magnification errors during acquisition or analysis. In our study we have not studied axial length as well as intraocular pressure due to limited time of examination. Strengths of this study include the use of the new generation Cirrus SD-OCT, novel analysis including intra- and interocular measurement.

## Conclusions

Normal GC-IPL topography in healthy children shows fairly large variations in average thickness between individuals. To increase sensitivity to detect pathologic GC-IPL thinning we suggest to look for differences between sector within eye and between eyes on an individual level since they are very small in healthy eyes.

To detect pathologic GC-IPL loss we suggest that asymmetry is considered as well as the difference between the average sector GC-IPL thickness compared to the minimum GC-IPL thickness.

## Data Availability

The datasets analyzed during the current study are available from the corresponding author on reasonable request.

## Abbreviations

Avg: Average
EXPRESS: Extremely Preterm Infants in Sweden Study
GA: Gestational age
GC-IPL: Ganglion cell + inner plexiform layer
I: Inferior
IH: Inferior hemisphere
IN: Inferonasal
IT: Inferotemporal
LE: Left eye
Min: Minimum
N: Nasal sectors
OCT: Optical coherence tomography
pRNFL: Peripapillary retinal nerve fiber layer
RE: Right eye
RTSD: Retrograde trans-synaptic degeneration
S: Superior
SD-OCT: Spectral domain technology
SD: Standard deviation
SH: Superior hemisphere
SN: Superonasal
ST: Superotemporal
T: Temporal sectors
VA: Visual acuity
